# Role of 2-Arachidonoyl-Glycerol and CB1 Receptors in Orexin-A-Mediated Prevention of Oxygen–Glucose Deprivation-Induced Neuronal Injury

**DOI:** 10.3390/cells9061507

**Published:** 2020-06-20

**Authors:** Letizia Palomba, Andrea Motta, Roberta Imperatore, Fabiana Piscitelli, Raffaele Capasso, Federica Mastroiacovo, Giuseppe Battaglia, Valeria Bruno, Luigia Cristino, Vincenzo Di Marzo

**Affiliations:** 1 Endocannabinoid Research Group, Institute of Biomolecular Chemistry (ICB), National Research Council (CNR), 80078 Pozzuoli (NA), Italy; fpiscitelli@icb.cnr.it (F.P.); raffaele.capasso@unina.it (R.C.); luigia.cristino@icb.cnr.it (L.C.); or vincenzo.di-marzo.1@ulaval.ca (V.D.M.); 2 Department of Biomolecular Sciences, University “Carlo Bo”, 61029 Urbino, Italy; 3 Institute of Biomolecular Chemistry, Consiglio Nazionale delle Ricerche, 80078 Pozzuoli, Italy; andrea.motta@icb.cnr.it; 4 Department of Science and Technology, University of Sannio, 82100 Benevento, Italy; rimperatore@unisannio.it; 5 Department of Agricultural Sciences, University of Naples Federico II, 80055 Portici, Italy; 6 IRCCS Neuromed, 86077 Pozzilli (IS), Italy; federica.mast@neuromed.it (F.M.); giuseppe.battaglia@neuromed.it (G.B.); valeria.bruno@neuromed.it (V.B.); 7 Department of Physiology and Pharmacology, University Sapienza, 00185 Roma, Italy; 8 Canada Excellence Research Chair on the Microbiome-Endocannabinoidome Axis, Université Laval, Quebec City, QC G1V 0A6, Canada

**Keywords:** endocannabinoids, orexin-A, oxygen–glucose deprivation, ischemia, radical oxygen species, neuronal cell death

## Abstract

Orexin-A (OX-A) protects the brain against oxidative stress-mediated ischemic injury. Since the endocannabinoid 2-arachidonoylglycerol (2-AG) and cannabinoid type-1 (CB1) receptors were previously shown to mediate some of the effects of OX-A exerted through the orexin-1 receptor (OX-1R), we investigated the involvement of 2-AG in OX-A-induced neuroprotection following oxygen and glucose deprivation (OGD) in mouse cortical neurons. OGD-induced reactive oxygen species (ROS) accumulation and neuronal death were prevented by both OX-A and arachidonyl-2′-chloroethylamide (ACEA), a synthetic CB1 receptor agonist, in a manner sensitive to OX-1R and CB1 receptor antagonists, SB334867 and AM251. OX-A stimulated 2-AG biosynthesis in cortical neurons. In neurons isolated from monoacylglycerol lipase (MAGL, a 2-AG hydrolyzing enzyme) null mice, 10-fold higher 2-AG concentrations were found and OGD failed to induce ROS production and cell death, whereas AM251 restored these noxious effects. OX-A-induced neuroprotection was mediated by the phosphoinositide-3-kinase/Akt (PI3K/Akt) survival pathway since both OX-A and ACEA induced phosphorylation of Akt and prevented OGD-induced cytochrome c release from the mitochondria, in a manner counteracted by SB334867 or AM251. Administration of OX-A reduced infarct volume and elevated brain 2-AG levels in a mouse model of transient ischemia. These results suggest that 2-AG and CB1 receptor mediate OX-A prevention of ischemia-induced neuronal apoptosis.

## 1. Introduction

Orexins (orexin-A and orexin-B; also known as hypocretins-1 and -2) are neuropeptides that are widely distributed in the central nervous system and peripheral tissues. However, orexin-producing neurons are localized only in the lateral hypothalamic area and nearby regions, with projection to numerous brain structures, and are regulated by many neurotransmitter systems [1,2,3,4]. Orexin-A (OX-A) and orexin-B (OX-B), derived from a single precursor prepro-orexin, act via two types of G-protein-coupled receptors with seven-transmembrane domain topology, the orexin-1 receptor (OX-1R) and orexin-2 receptor (OX-2R) [5]. The anatomical architecture of orexin neurons and the wide distribution of orexin receptors appear to be essential for the multiple functions of these neuropeptides. Orexins play an important role in many physiological aspects of mammalian life, including the control of food intake and sleep–wake behavior [5,6], as well as, among many others, the protection of neurons against oxidative stress and ischemic brain injury [7,8,9]. Brain ischemia is a neurological disorder that occurs when a part of the brain is deprived of oxygen and glucose, with subsequent neuronal cell death mainly due to oxygen and glucose deprivation (OGD)-mediated oxidative stress [10]. Radical oxygen species (ROS) are generated to a small extent during ischemia, while a far greater production occurs after reintroduction of oxygen during reperfusion.

Recent studies have shown that OX-A activation of OX-1R stimulates the biosynthesis of 2-arachidonoylglycerol (2-AG) [11,12], the most abundant endocannabinoid (EC) in the brain [13,14,15]. This lipid mediator is involved in several physiological and pathological aspects of the CNS (see [16] for review), including neuronal survival after cerebral ischemic insult [17]. Indeed, OX-1R-induced 2-AG biosynthesis and subsequent activation of cannabinoid type-1 (CB1) receptors, the main target for ECs in the brain, underlies several effects of OX-A in the brain [12,18]. Therefore, in this study, an in vitro model of oxygen–glucose deprivation (OGD) was used to mimic some pathological aspects of ischemia. In particular, we investigated if the neuroprotective effect of OX-A on OGD-induced ROS formation in primary cultures of mouse cortical neurons was mediated by 2-AG biosynthesis and subsequent CB1 activation, and through what molecular mechanisms. Moreover, we validated the neuroprotective effect OX-A in vivo by using an animal model of brain ischemia.

## 2. Materials and Methods

### 2.1. Primary Cortical Neuron Cultures

Primary cultures of mouse cortical neurons, derived from neonatal or 1-day-old C57BL/6 (Charles River) or monoacylglycerol lipase (MAGL) null mice (a kind gift by the Institute of Molecular Biosciences, University of Graz, Graz, Austria), were acquired as described [19]. Briefly, the cerebral cortex was quickly separated and mechanically dispersed in Ca^2+^- and Mg^2+^-free buffered Hanks’ balanced salt solution. Then, tissues were dissociated enzymatically (0.125% trypsin solution, 37 °C for 20 min) and mechanically. Cells were inoculated at a density of 2 × 10^4^ cells/cm^2^ on polylysine-coated coverslips and grown at 37 °C in Neurobasal medium supplemented with 2% B27, 0.5 mM L-glutamine, penicillin (50 U/mL), and streptomycin (50 µg/mL), gassed with an atmosphere of 95% air and 5% CO_2_. Cells were used between 6 and 8 d in vitro. More than 80% of primary cultured cells were positive for neuronal marker NeuN antibodies, determined by immunocytochemistry (data not shown).

### 2.2. Oxygen–Glucose Deprivation

Primary cortical neurons were subjected to OGD as described elsewhere [20]. Briefly, the Neurobasal culture medium was replaced with oxygen/glucose-free balanced salt solution (BSS, in mmol/l: 116 mM NaCl, 5.4 mM KCl, 0.8 mM MgSO_4_, 1 mM NaH_2_PO_4_. ^2^H_2_O, 262 mM NaHCO_3_, 1.8 mM CaCl_2_, pH 7.2, <0.1% O_2_), which was previously saturated with 95% N_2_/5% CO_2_ at 37 °C. Neurons were transferred to an anaerobic chamber (Plas-Labs, Lansing, MI, USA) equilibrated for 10 min with a continuous flux of gas (95% N_2_/5% CO_2_). Cells were taken out of the chamber after 60 min and the oxygen/glucose-free BSS was replaced with Neurobasal medium and, finally, transferred to the regular cell culture incubator for increasing time intervals in the normoxic conditions. Control neurons were incubated in complete Neurobasal medium in a normoxic incubator for the same period of time.

### 2.3. Measurement of Reactive Oxygen Species (ROS)

ROS formation was assayed using dihydrorhodamine 123 (DHR) as described by Palomba et al. [19]. Briefly, primary cortical neurons, loaded with DHR (10 µM for 20 min), were processed as detailed in the figure legends and analyzed with a Leica DMI6000 fluorescence microscope equipped with a Leica DFC320 cooled digital CCD camera (Leica Microsystems, Milan, Italy). The excitation and emission wavelengths were 488 and 515 nm, respectively. Pictures were collected with exposure times of 100–400 ms, digitally acquired and analyzed for fluorescence determination at the single cell level with the Metamorph Imaging Software (Leica MetaMorph© AF). Mean fluorescence values were determined by averaging the fluorescence values of at least 50 cells/treatment condition/experiment.

### 2.4. Cellular Viability Assay

The decrease in 3-(4,5-dimethylthiazol-2-yl)-2,5-diphenyltetrazolium bromide (MTT) was assessed as a measure of cell viability [21]. Briefly, after treatments, the MTT solution (5 mg/mL) was added to the culture and incubated for an additional 4 h. Formazan produced by the neurons was measured using an ELISA 96-well plate reader (Bio-Rad Laboratories, Hercules, CA, USA) at 490 nm of the absorbance.

### 2.5. Immunocytochemistry

Primary cortical neurons were fixed for 20 min with paraformaldehyde (4%; *v*/*v*), washed with phosphate-buffered saline (PBS) and blocked in PBS-containing bovine serum albumin (BSA) (2%, *w*/*v*). A goat anti-OX-1R antibody (Santa Cruz, CA, USA), a rabbit anti-CB1R (anti C terminus 461–472; Abcam) or a mouse anti-NeuN (Abcam) antibody were used as primary antibody. After 18 h at 4 °C, the neurons were rinsed and exposed to a fluorescein isothiocyanate-conjugated secondary antibody for 2 h in the dark. Stained cells were analyzed with a Leica DMI6000 fluorescence microscope supplied with a Leica DFC320 cooled digital CCD camera (Leica Microsystems).

### 2.6. Western Blot Analysis

After treatments, primary neurons were processed to obtain whole cell lysates and mitochondrial and cytosolic fractions, as described [22]. Western blot analysis was next performed. Protein extracts were subjected to electrophoresis on a 12% polyacrylamide gel and transferred to Polyvinylidene fluoride (PVDF) membranes. Membranes were blocked with non-fat dry powdered milk for 2 h and incubated overnight at 4 °C with antibodies against Akt, Akt phosphorylated at serine 473, Bcl-2, or cytochrome c (1:500; Calbiochem), whereas incubation was for 1 h at room temperature with the horseradish peroxidase -conjugated goat α-rabbit secondary antibody (1:1000; Biorad). A monoclonal anti-β-actin antibody (1:4000; Sigma-Aldrich) was used as to assess reference protein expression. Detection was performed using chemiluminescence (Clarity ECL; Biorad). Images were analyzed on a ChemiDoc station with ImageJ software.

### 2.7. EC Measurements

After treatments, mice were killed by cervical dislocation, the brains removed and the cerebral cortex rapidly (<5 min) dissected. Tissue samples were homogenized in 5 vol chloroform/methanol/Tris·HCl 50 mM (2:1:1 by volume), containing 50 pmol of d5-2-arachidonoylglycerol (d5-2-AG) and 5 pmol of d8-anandamide (d8-AEA) as internal standards. Homogenates were centrifuged at 13,000× *g* for 16 min (4 °C); the aqueous phase plus debris were collected and extracted four times with 1 vol chloroform. The lipid-containing organic phases were pooled, dried, and pre-purified by open-bed chromatography on silica columns eluted with increasing concentrations of methanol in chloroform. Fractions for EC measurement were obtained by eluting the columns with 9:1 (by volume) chloroform/methanol and then analyzed by liquid chromatography-atmospheric pressure chemical ionization-mass spectrometry (LC-APCI-MS). LC-APCI-MS analyses were carried out in the selected ion monitoring mode, using *m/z* values of 384.35 and 379.35 (molecular ions +1 for deuterated and undeuterated 2-AG) and 347.5 and 355.5 (molecular ions +1 for deuterated and undeuterated AEA). Values are expressed as pmol per mg of wet tissue extracted.

For EC levels in neurons, after treatments, primary cortical neurons and their supernatants were collected, homogenized, and analyzed as indicated above for tissues. EC levels were normalized per mL of cell + medium. Each sample contained 0.5 × 10^5^ cells/mL in 2 mL. In some experiments, the number of ECs in neurons was measured after stimulation with OX-A in the presence or absence of the diacylglycerol lipase (DAGL) inhibitor, O-7460 [23].

### 2.8. Animals

Male C57BL6 mice (20–24 g) purchased from Charles River (Calco, Italy) were used for the induction of permanent or transient focal ischemia. Mice were housed under standard conditions with a 12 h light/dark cycle and food and water ad libitum. Studies were carried out in accordance with the National Guidelines for Animal Use (Italian Parliament DL.116/92) and approved by the Italian Ministry of Health. All efforts were made to minimize the potential sufferance and discomfort of animals and their number.

### 2.9. Transient Focal Ischemia in Mice

Mice (10-weeks old, 22 to 24 g body weight) were treated with isoflurane (3% for induction and 2% for maintenance) in N_2_O/O_2_ (70:30). A rectal temperature probe associated with a heating pad was used to maintain body temperature at 37 °C throughout the surgical period (up to 60 min after the induction of focal ischemia).

For induction of transient middle cerebral artery occlusion (MCAO), a silicon-coated filament (200 µm) was inserted into the internal carotid artery until it blocked the origin of the middle cerebral artery (MCA). Cerebral blood flow was routinely measured in mice by taking away the skin over the right hemisphere and fixing a flexible optical filament by instant glue on the skull in correspondence to a major branch of the MCA on the right side of the skull (4 mm from the midline and 2 mm posterior to the bregma). The optical filament was united to a laser Doppler flow meter (PeriFlux System; Perimed, Cuggiono, Italy) for the assessment of cerebral blood flow. Cerebral blood flow was determined throughout the surgical procedure when the animal was under deep anesthesia, including 30 min before, 45 min of occlusion, and 20 min after MCAO. Monofilament placement was established by a reduction of cerebral blood flow (>80% basal value) by laser Doppler. Mice with adequate occlusion were included in the study. These mice had a (i) regional blood flow reduction > 80%, (ii) a sustained reduction of regional blood flow throughout the occlusion time, and (iii) a complete rescue of regional blood flow within 5 min after removal of monofilament. Sham-operated mice were subjected to the same anesthesia and surgical procedure, except for MCAO. After surgery, all mice were positioned in an incubator (Compact incubator, Thermo Scientific, AHSI, Bernareggio, Italy) at 37 °C for 120 min, and then brought back to their home cages. Ischemic mice were injected with 0.5 mL of 5% glucose in Krebs subcutaneous every 24 h.

Mice undergoing to transient MCAO were injected intraperitoneal (ip.) with either saline or orexin-A (40 µg/kg, dissolved in saline) 30 min before MCAO. Mice were killed 48 h after ischemia.

### 2.10. Histological Analysis and Assessment of the Infarct Volume

Brains were cut and fixed in Carnoy’s solution, embedded in paraffin, and sectioned at 10 µm. Sections were deparaffinized and processed for staining with thionin (Nissl staining for histologic assessment of neuronal degeneration). The analysis was executed on sections regularly spaced every 550 µm through the extension of the ischemic region. The infarct volume was determined by integrating the cross-sectional area of damage on each section and the distance between the various levels. In each stained section, the necrotic area and the total area of the ipsilateral hemisphere were identified and outlined at a magnification of 2.5× and analyzed using Scion Image software (NIH, Bethesda, MD, USA). The infarct volume (V) and the total ipsilateral hemisphere volume were calculated by the following formula: V = S(*Ai* × *TS* × *n*), where *Ai* is the ischemic area measured at the ith section, *TS* is the section thickness (10 µm), and *n* is the number of sections between two adjacent levels. Infarct volumes are expressed as the % hemisphere volume.

### 2.11. Statistical Analysis

Data are expressed as mean ± SEM unless otherwise indicated and were analyzed using the GraphPad Prism 6 software, version 6.05 (GraphPad, Inc., San Diego, CA, USA). Statistical differences among groups were determined by either Student’s *t*-test or two-way ANOVA followed by Bonferroni test. The data were normally distributed. A level of confidence of *p* < 0.05 was taken for statistical significance.

## 3. Results

### 3.1. OX-A Prevents OGD-Induced ROS Formation in Primary Cultures of Mouse Cortical Neurons

**Figure 1:**
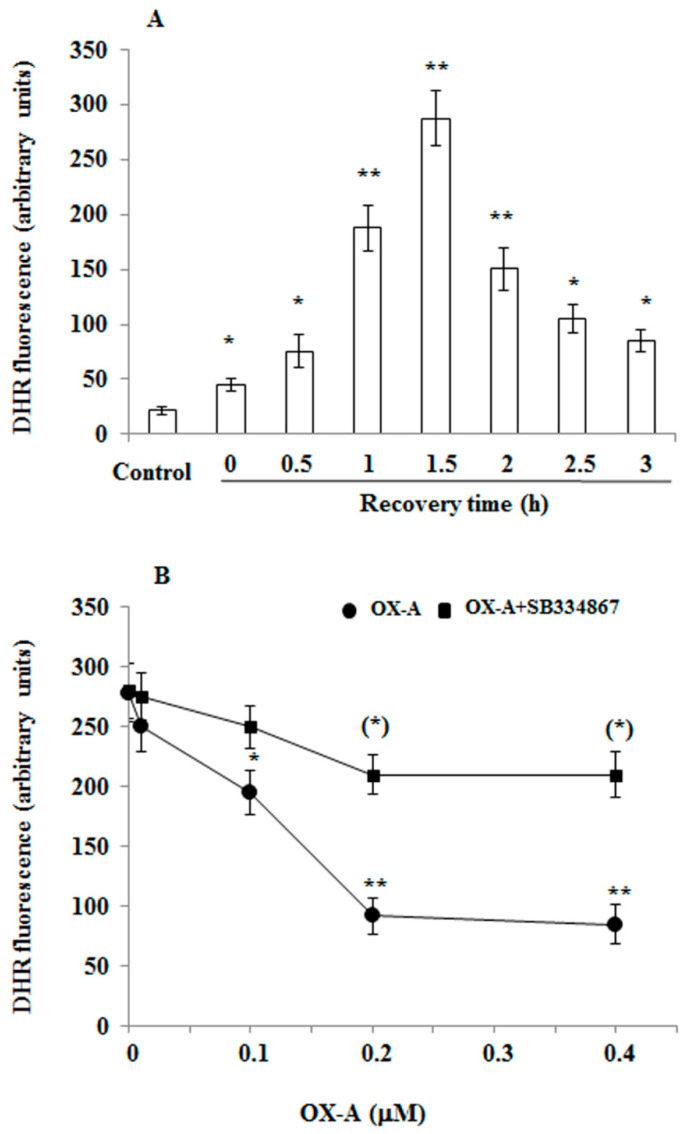
Orexin-A (OX-A) prevents oxygen–glucose deprivation (OGD)-induced reactive oxygen species (ROS) formation in cortical primary neurons. (**A**) Dihydrorhodamine 123 (DHR)-loaded primary neurons were exposed to OGD for 60 min followed by reoxygenation for increasing time intervals or were maintained under normoxic conditions. After treatment, the cells were observed with a Leica DMI6000 fluorescence microscope equipped with a Leica DFC320 cooled digital CCD camera (Leica Microsystems). The resulting images were analyzed to quantify the mean fluorescence of individual cells using the Metamorph Imaging Software (Leica MetaMorph AF). Results are expressed as arbitrary units and represent the mean ± SEM calculated from three to five separate experiments, each performed in duplicate. * *p* < 0.05; ** *p* < 0.01 vs. normoxic-treated cells (one-way ANOVA followed by Bonferroni test). (**B**) DHR-loaded primary neurons were treated with the different concentration of OX-A for 30 min in the absence or presence of SB334867 (10 µM, added 15 min before OX-A) and, finally, exposed to OGD for 60 min. After treatment, the cells were analyzed with a fluorescence microscope as described in (A). Results expressed as arbitrary units represent the mean ± SEM calculated from three to five separate experiments, each performed in duplicate. * *p* < 0.05, ** *p* < 0.001 vs. OGD-treated cells; (**) *p* < 0.01 vs. OX-A-treated cells (one-way ANOVA followed by Bonferroni test).

It is well known that ROS formation is observed during ischemia/reperfusion. While only a small amount of ROS is generated during ischemia, far greater production occurs during reperfusion. Since OX-A is protective against oxidative stress [8,24,25], we used OGD-induced injury, an in vitro model of cerebral ischemia/reperfusion, to determine if OX-A can offer neuroprotection by preventing ROS formation. After 60 min exposure of primary mouse cortical neurons to OGD followed by increasing time interval in the normotoxic condition, ROS formation, assessed with DHR, a cell-permeable fluorogenic probe, was detected with maximum effect at 1.5 h (Figure 1A). This effect was prevented by pre-treatment with OX-A (30 min) in a dose-dependent manner (Figure 1B), and was mediated by OX-1R, since the protective effect of the neuropeptide was blocked by SB334867 (10 µM, added 15 min before OX-A), a specific OX-1R antagonist (Figure 1B).

### 3.2. OX-A Prevents OGD Injury by Inducing 2-AG Biosynthesis and Subsequent Activation of CB1 Receptors

Recently, we reported that in hypothalamic neurons OX-A stimulates the biosynthesis of 2-AG [12], the most abundant EC in the brain [13,14,15], hydrolyzed primarily by MAGL [26]. This effect of OX-A was observed here also in primary cultures of mouse cortical neurons, where 2-AG levels were increased after treatment with the neuropeptide (0.2 µM for 30 min; Table 1). OX-A-induced elevation of 2-AG concentrations was prevented by O-7460, a selective inhibitor of DAGLs, the enzymes catalyzing 2-AG formation.

**Figure 2:**
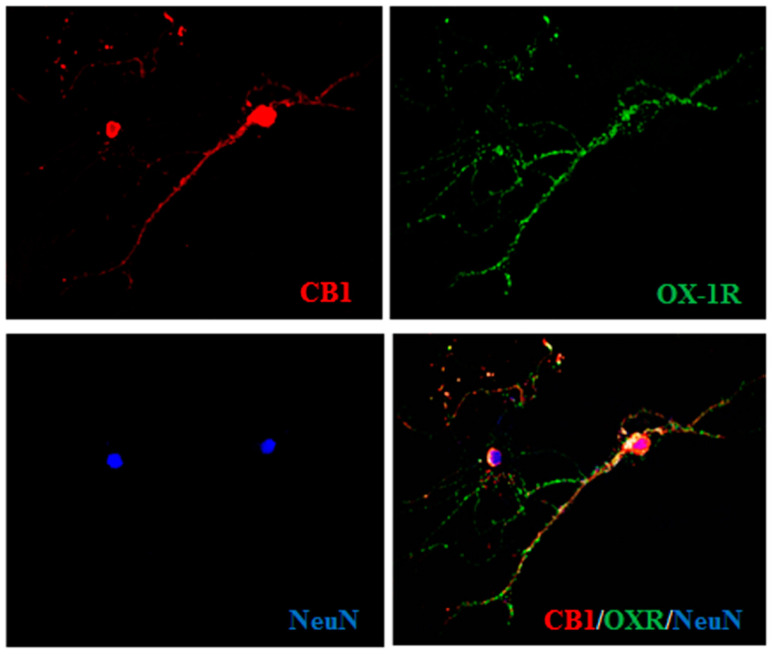
Expression of cannabinoid type-1 (CB1) and orexin-1 receptor (OX-1R) in primary cortical neurons. Representative micrographs of immunocytochemical staining of the CB1 (red signal) or OX-1R (green signal) receptors in a primary culture of cortical neurons. Neuronal-specific nuclear protein (NeuN) antibody (blue signal) was used as marker of neuronal cells. Scale bar: 20 µm.

**Figure 3:**
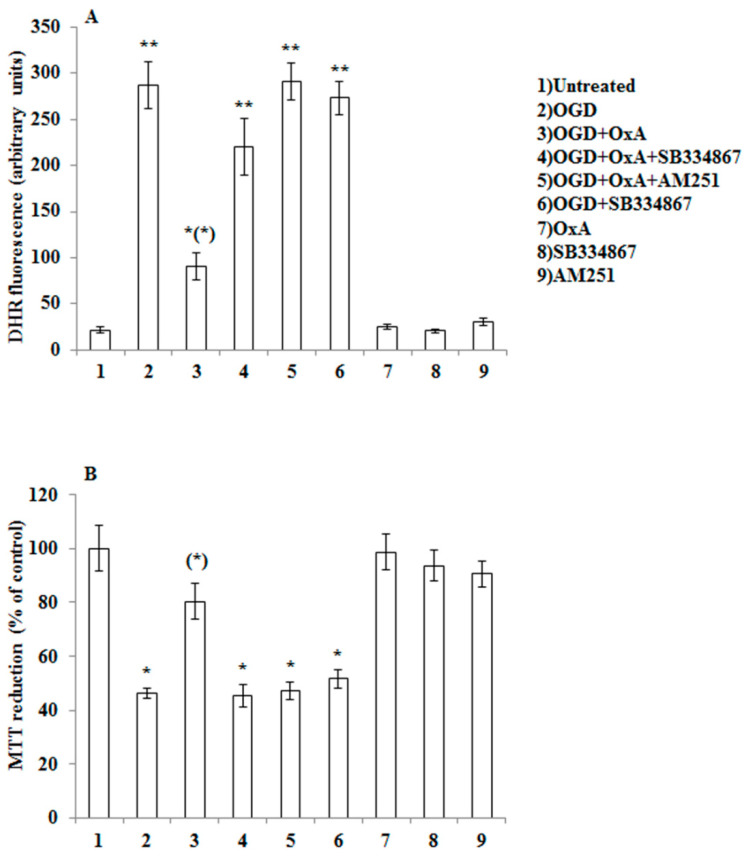
OX-A prevents OGD-mediated ROS toxicity in primary cortical neurons. (**A**) DHR-loaded cells were incubated with AM251 (0.5 µM) or SB 334867 (10 µM) for 15 min, exposed to OX-A (0.2 µM) for an additional 30 min and, finally, subjected to OGD for 60 min. The cells returned to the incubator under normoxic conditions for 1.5 h before analysis with a fluorescence microscope as described in Figure 1. Results expressed as arbitrary units represent the mean ± SEM calculated from three to five separate experiments, each performed in duplicate. * *p* < 0.05, ** *p* < 0.001 vs. control cells; (*) *p* < 0.001 vs. OGD-treated cells (one-way ANOVA followed by Bonferroni test). (**B**) Neurons were treated as described in (A) and after 20 h in the incubator under normoxic conditions were analyzed for cell viability using the 3-(4,5-dimethylthiazol-2-yl)-2,5-diphenyltetrazolium bromide (MTT) assay. Results are expressed as the percentage of viable cells detected following OGD compared to control normoxic plates. Results represent the mean ± SEM of three to five separate experiments, each performed in duplicate. * *p* < 0.001 vs. control cells; (*) *p* < 0.001 vs. OGD-treated cells (one-way ANOVA followed by Bonferroni test).

**Figure 4:**
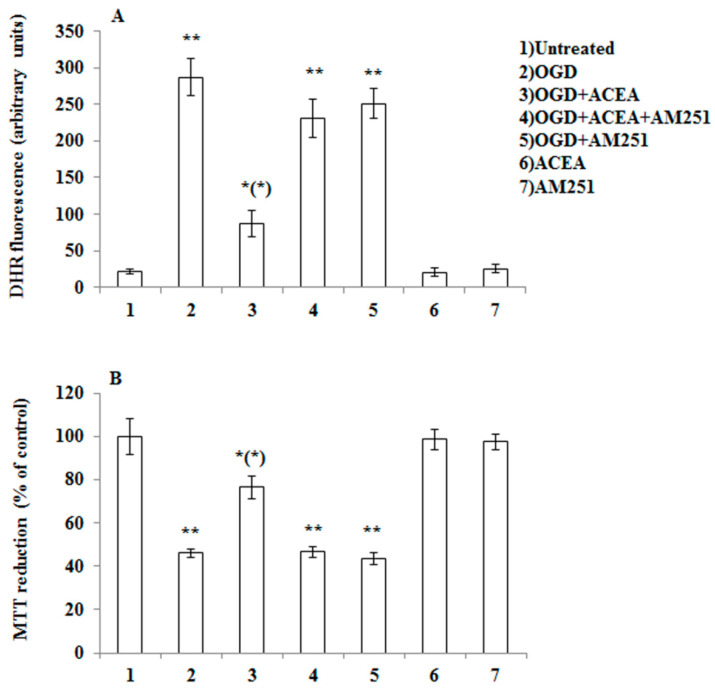
CB1 stimulation with arachidonyl-2′-chloroethylamide (ACEA) prevents OGD-induced ROS formation as well as toxicity in primary cortical neurons. (**A**) DHR-loaded cells, were incubated for 15 min with AM251 (0.5 µM), exposed to ACEA (0.5 µM) for an additional 15 min and, finally, subjected to OGD (60 min). After 1.5 h incubation under normoxic conditions, the cells were analyzed with a fluorescence microscope as described in Figure 1. Results expressed as arbitrary units represent the mean ± SEM calculated from three to five separate experiments, each performed in duplicate. * *p* < 0.05, ** *p* < 0.001 vs. control cells; (*) *p* < 0.001 vs. OGD-treated cells (one-way ANOVA followed by Bonferroni test). (**B**) Neurons were treated as in (A), exposed to OGD for 60 min, and after 20 h under normoxic conditions, were analyzed for cell viability using the MTT assay. Results represent the mean ± SEM of three to five separate experiments, each performed in duplicate. * *p* < 0.05, ** *p* < 0.001 vs. control cells; (*) *p* < 0.01 compared to OGD-treated cells (one-way ANOVA followed by Bonferroni test).

In order to provide evidence for the intermediacy of 2-AG and CB1 receptors in OX-A prevention of OGD-induced ROS formation and subsequent neuron death, we adopted a dual experimental strategy: (1) we exposed to OGD primary neurons pre-treated with arachidonyl-2′-chloroethylamide (ACEA), a selective CB1 receptor agonist; and (2) we performed experiments in primary cortical neurons isolated from MAGL null mice, which are characterized by higher 2-AG levels (Table 1) [27,28]. We first confirmed the expression of both CB1 and OX-1R by immunocytochemistry in mouse primary cortical neurons (Figure 2). Next, as shown in Figure 3A, we demonstrated that the protective effect of OX-A (0.2 µM) on OGD-induced ROS formation was abolished by treating the cells with AM251 (0.5 µM; added 15 min before OX-A), a CB1 receptor antagonist/inverse agonist, as well as by SB334867 (10 µM, added 15 min before OX-A). Additionally, the cell death, determined by the MTT assay, induced by 1 h exposure to OGD followed by 24 h of reoxygenation, was also reduced by pre-exposure to OX-A (0.2 µM for 30 min), in a manner again sensitive to both SB334867 (10 µM, added 15 min before OX-A) and AM251 (0.5 µM, added 15 min before OX-A) (Figure 3B). In the same experimental condition, ACEA (0.5 µM), added 15 min before OGD, prevented both ROS formation and cell death in a manner reversed by AM251 (0.5 µM, added 15 min before ACEA) (Figure 4A,B).

**Figure 5:**
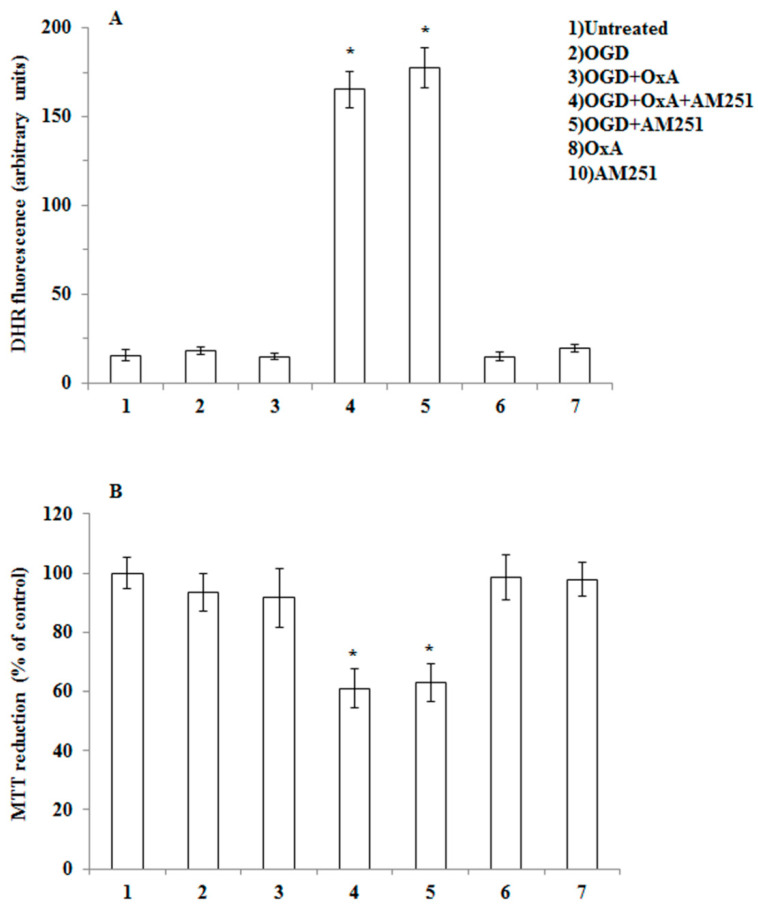
OGD treatment fails to induce ROS formation and toxicity in primary cortical neurons isolated from MAGL null mice. (**A**) Primary neurons isolated from MAGL null mice, loaded with DHR, were incubated with AM251 (0.5 µM, 15 min), exposed to OX-A (0.2 µM) for an additional 30 min and, finally, subjected to OGD for 60 min. After 1.5 h incubation under normoxic conditions, the cells were analyzed with a fluorescence microscope as described in Figure 1. Results expressed as arbitrary units represent the mean ± SEM calculated from three to five separate experiments, each performed in duplicate. * *p* < 0.0001 vs. OGD-treated cells (one-way ANOVA followed by Bonferroni test). (**B**) Neurons were treated as in (**A**), exposed to OGD for 60, and after 20 h under normoxic conditions, analyzed for cell viability using the MTT assay. Results represent the mean ± SEM of three separate experiments, each performed in duplicate. * *p* < 0.01 vs. OGD-treated cells (one-way ANOVA followed by Bonferroni test).

To provide additional evidence for the involvement of CB1 in OX-A-induced neuroprotection, experiments were performed in primary neurons isolated from MAGL null mice (MAGL^-/-^), where the levels of 2-AG were found here to be ~10-fold higher compared to primary neurons from wild-type mice (Table 1). As shown in Figure 5, exposure of neurons from MAGL^-/-^ mice to OGD did not result in ROS formation (Figure 5A) and subsequent cell death (Figure 5). This lack of effect was most likely due to increased tonic activation of CB1 induced by 2-AG, since AM251 treatment restored both OGD-induced ROS formation and cell death (Figure 5). These data strongly suggest that 2-AG mediates the neuroprotection induced by OX-A, at least in primary cortical neurons exposed to OGD insult.

### 3.3. OX-A Counteracts OGD-Induced Inhibition of the PI3K/Akt Signaling

**Figure 6:**
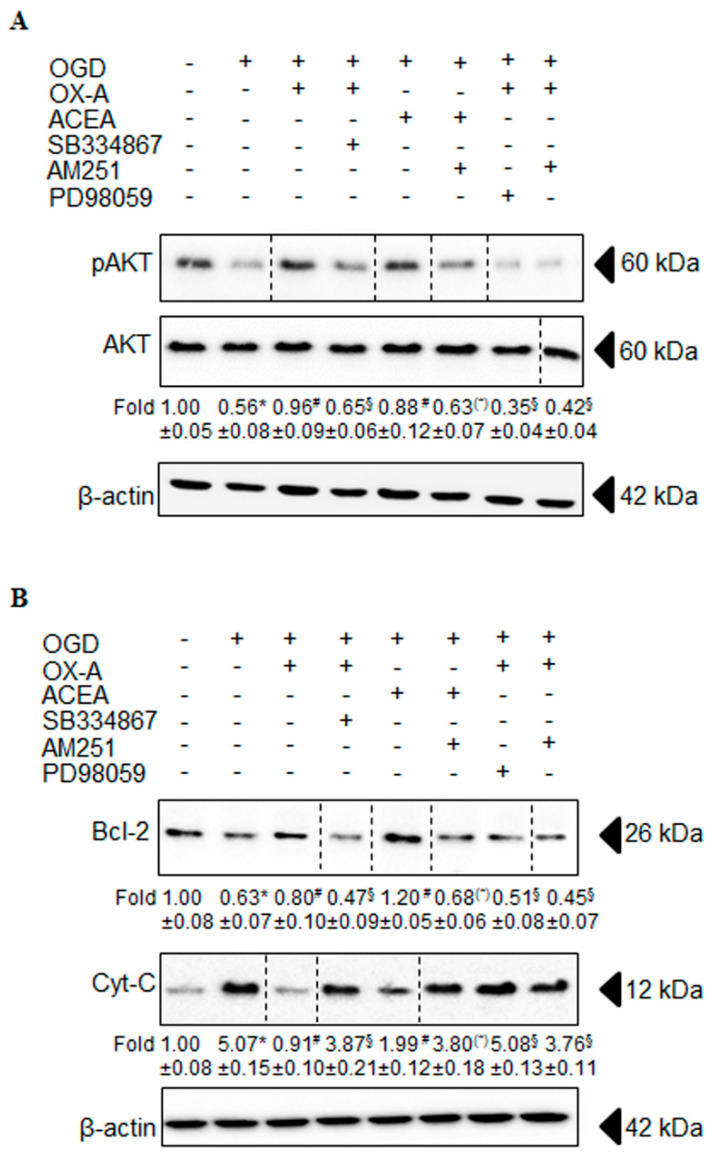
Effect of OX-A or ACEA on OGD-induced apoptosis in primary cortical neurons. (**A**) Neurons were incubated with AM251 (0.5 µM), SB 334867 (10 µM) or PD98059 (50 µM) for 15 min, exposed to OX-A (0.2 µM) for an additional 30 min and, finally, subjected to OGD for 60 min. The cells returned to the incubator under normoxic conditions for 20 h prior to analyzing the total cell extract with Western blotting using anti-phospho-Akt ^serine 473^ or AKT antibodies. Fold data represent the mean ± SEM of three separate experiments, each performed in duplicate, normalized to the total proteins present in the extract of control cells. (**B**) Cells, treated as in (**A**), returned to the incubator under normoxic conditions for 20 h prior to isolating the mitochondrial and cytosolic fractions. Cytosolic fractions were finally processed for Western blot analysis using cytochrome c or Bcl-2 antibodies. Fold data represent the mean ± SEM of three separate experiments, each performed in duplicate, normalized to the total proteins present in the cytosolic fractions of control cells. Blots shown are representative of three separate experiments with similar outcomes. The β-actin bands confirm that similar amounts of proteins were loaded on the gel for each sample. * *p* < 0.01 vs. untreated cells; ^#^ *p* < 0.01 vs. OGD-treated cells; ^§^ *p* < 0.01 vs. OGD/OX-A-treated cells; (*) *p* < 0.01 vs. OGD/ACEA-treated cells (one-way ANOVA followed by Bonferroni test).

Since neuronal cells exposed to OGD insult undergo apoptotic death [29,30] mediated by the phosphatidylinositol-3-kinase (PI3K)/Akt and ERK1/2 pathways [31,32], we investigated the effect of OX-A on these pathways. Primary cortical neurons were exposed to OGD (1 h followed by 24 h of reoxygenation) in the absence or presence of OX-A (0.2 µM added 30 min before OGD), followed by protein extraction and Western blot analysis. OX-A treatment increased significantly the levels of p-Akt compared to treatment with OGD alone (Figure 6A). This response was prevented by pre-incubation with either SB334867 (10 µM, added 15 min before OX-A) or AM251 (0.5 µM; added 15 min before OX-A). Under the same conditions, ACEA (0.5 µM added 15 min before OGD) also prevented the decrease of p-Akt levels induced by OGD in a manner sensitive to AM251 (0.5 µM; added 15 min before ACEA). Additionally, pretreatment of cells with PD98059 (50 µM), an ERK1/2 phosphorylation inhibitor, blocked the OX-A-mediated increase of p-Akt (Figure 6A), suggesting that OX-A may inhibit the insult induced by OGD via the activation of the PI3K/Akt and ERK1/2 cell survival pathways [33,34].

### 3.4. Effects of OX-A on Bcl-2 Expression and Cytochrome c Release

Bcl-2 inhibits apoptosis by preventing mitochondrial membrane depolarization [35] and, consequently, cytochrome c release from the mitochondria into the cytosol [35]. Since OGD induces cytochrome c release from the mitochondria into the cytosol [36] by interfering with Bcl-2 expression, we investigated the possible effect of OX-A on Bcl-2 levels in the cytosol. We found that OGD decreased Bcl-2 protein levels, whereas OX-A treatment prevented this effect in a manner sensitive to both SB334867 (10 µM; added 15 min before OX-A) and AM251(0.5 µM; added 15 min before OX-A) (Figure 6B). Additionally, OGD induced cytochrome c release and this effect was also blocked by OX-A, in a manner reverted again by either SB334867 or AM251 treatment (Figure 6B).

### 3.5. Effects of OX-A in MCAO-Induced Focal Cerebral Ischemia

**Figure 7:**
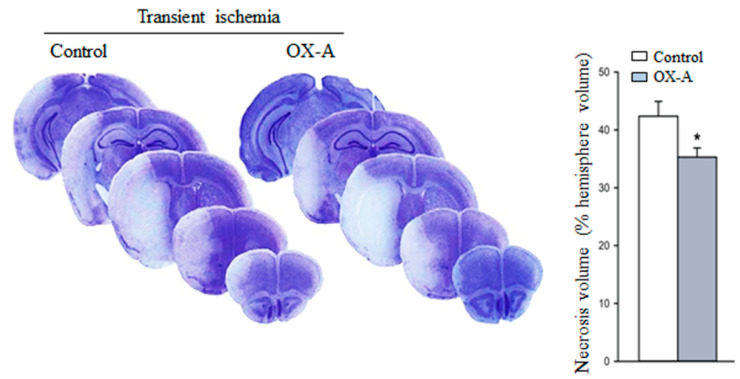
Systemic injection of OX-A reduces necrosis volume in the middle cerebral artery occlusion model of transient focal ischemia. Representative images of brain Nissl staining of male mice subjected to transient MCAO and treated i.p with saline or OX-A (40 µg/kg, injected 30 min before the onset of ischemia). Mice were killed 48 h after MCAO. Values of the infarct volume are mean ± SEM; *n* = 6 mice per group. Values were calculated by integrating the cross-sectional area of damage at each bregma level and the distances between the various levels and are expressed as the % necrotic volume of the entire right cerebral hemisphere. * *p* < 0.05 (Student’s *t*-test).

To determine whether OX-A treatment may attenuate the damage induced by ischemia in vivo, we examined the effect of the neuropeptide in MCAO mice. We carried out experiments in mice undergoing transient MCAO treated systemically with OX-A. The drug was injected intraperitoneally 30 min before MCAO at a dose of 40 µg/kg, which was shown to be fully effective in in vivo studies [18]. Treatment with OX-A significantly reduced the infarct volume (Figure 7).

To provide evidence for 2-AG involvement in OX-A induced neuroprotection in vivo, we analyzed the levels of ECs in the cerebral cortex of mice 48 h after transient MCAO with or without OX-A administration. Transient MCAO resulted in the reduction of 2-AG levels in mouse cerebral cortex, and OX-A treatment prevented this effect (Table 2). Interestingly, OX-A also increased the levels of the other EC, AEA, which instead was unaffected by transient MCAO per se.

## 4. Discussion

OX-A, a neuropeptide produced by the lateral hypothalamus, has a protective effect on cerebral ischemia-induced cell death [7,9]. Accordingly, in the present study we found that treatment with OX-A resulted in protection from ischemia/reperfusion injury in both the in vitro and in vivo models used. The mechanism by which OX-A prevents ischemia-induced neuronal death, however, is currently unclear. The data presented here suggest that such a mechanism involves the sequential biosynthesis of the most abundant brain EC, 2-AG, the activation of CB1 receptors, the triggering of the ERK1/2-p-Akt survival cascade, and the subsequent inhibition of ischemia-induced neuronal apoptosis.

It is well known that ECs, in particular 2-AG, are involved in neuroprotection from cerebral ischemia-induced cell death [16]. Recently, it has emerged that OX-A, through OX-1R activation, stimulates 2-AG biosynthesis via the phospholipase C-diacylglycerol lipase α (DAGLα) route [11,12,18]. Therefore, we hypothesized an involvement of 2-AG in the neuroprotection afforded by OX-A, and accordingly we found here that the neuropeptide stimulates 2-AG biosynthesis also in a primary culture of mouse cortical neurons. Consistent with the working hypothesis, we also found that OGD-induced ROS formation and the subsequent cell death in primary cerebral neurons was prevented by OX-A in a manner sensitive to not only SB334867, a specific OX-1R antagonist, but also AM251, a CB1 receptor antagonist/inverse agonist. The 2-AG-mediated mechanism of action of OX-A was further confirmed using primary neurons isolated from MAGL null mice, where the levels of 2-AG were much higher compared to primary neurons from wild type mice. As expected, OGD failed to induce ROS formation and cell death in these neurons, most likely due to their increased tone of 2-AG-mediated CB1 activation, since CB1 antagonism with AM251 restored both OGD-induced ROS formation and cell death.

Studies have shown that OGD-induced apoptotic cell death [29,30] occurs by the inhibiting PI3K/Akt and ERK1/2 pathways [31,32], and that neuroprotective effects of both OX-A- [37,38,39] and ECs [40,41] were dependent on the activities of these two pathways. We showed for the first time that the effect of OX-A on OGD-induced apoptotic cell death is mediated by CB1 activation. In fact, Akt phosphorylation and subsequent activation were prevented by AM251. We demonstrated that the anti-apoptotic protein Bcl-2 is a target for OX-A-induced neuroprotection, as OGD decreased the cytosolic levels of this protein and OX-A increased them back to control levels. Once again, the latter effect was prevented by the CB1 antagonist.

It well known that the Bcl-2 family proteins prevent apoptosis by regulating the permeability of the mitochondrial membrane [42] and cytochrome c release from the mitochondria [43]. The latter effect, via interaction with Apaf-1, leads to activation of caspase-9 and -3 [44,45,46] and, hence, to the apoptotic cascade. In agreement with previously reported data [36,47,48], we found that cytochrome c was translocated from the mitochondria to the cytosol after OGD also in primary cortical neurons, and showed that this effect was prevented by OX-A. The observation that AM251 was able to prevent this anti-apoptotic effect of OX-A suggests again the involvement of CB1 in OX-A-induced neuroprotection.

Finally, using the transient MCAO models for stroke, we demonstrated that in vivo administration of OX-A significantly reduced infarct volumes. Once again in agreement with the role of 2-AG/CB1 signaling in OX-A action, we found that OX-A increased 2-AG levels in the cerebral cortex of mice subjected to the transient ischemic insult, which per se reduced the tissue concentrations of this EC. Interestingly, OX-A, but not transient MCAO itself, also enhanced the cortical levels of the other EC, AEA. This effect, which may have contributed to the neuroprotection afforded by the neuropeptide, could be due to restoration of CB1 signaling by 2-AG, since it has been reported that CB1 activation can indirectly enhance AEA levels in the brain and peripheral tissues [49,50].

In conclusion, the results reported in this study demonstrate the role of 2-AG and CB1 receptors in OX-A-induced prevention of the neuronal damage caused by ischemia. These findings provide a basis for further studies to discover a new potential therapeutic approach in the clinical treatment of brain ischemic injury.

## Figures and Tables

**Table 1:** 2-Arachidonoylglycerol (2-AG) and anandamide (AEA) levels in primary cortical neurons isolated from C57BL/6 or monoacylglycerol lipase (MAGL) null mice.

	2-AG (pmol/mL)	AEA (pmol/mL)
**Wild type**		
Untreated	1.30 ± 0.50	0.06 ± 0.007
OX-A	241 ± 66.8 **	0.08 ± 0.030
OX-A+ O-7460	2.99 ± 2.64	0.07 ± 0.010
**MAGL−/−**		
Untreated	11.23 ± 0.54 *	0.04 ± 0.01

Endogenous levels of 2-AG or AEA were quantified by liquid chromatography-atmospheric pressure chemical ionization-mass spectrometry (LC-APCI-MS) in primary culture of cortical neurons isolated from C57BL/6 or MAGL null mice. In some experiments, primary neurons were treated with Ox-A (0.2 µM), with or without O-7460 (10 µM). 2-AG levels are normalized per volume of cells (each sample contains 0.5 × 10^5^ cells/2 mL). Results represent mean ± SEM of three separate experiments, each performed in duplicate. ** *p* < 0.0001 vs. wild type untreated cells; * *p* < 0.001 vs. wild type untreated cells (one-way ANOVA followed by Bonferroni test).

**Table 2:** 2-AG and AEA levels in mouse cerebral cortex after transient focal ischemia.

	2-AG (pmol/g Wet Tissue Weight)	AEA (pmol/g Wet Tissue Weight)
Sham	1.12 ± 0.27	0.064 ± 0.015
Transient ischemia	0.39 ± 0.19 *	0.058 ± 0.014
Transient ischemia + OX-A	0.70 ± 0.11 ^#^	0.088 ± 0.003 *^#^

Endogenous levels of 2-AG or AEA were quantified by LC-APCI-MS in the cerebral cortex of mice subjected to transient focal ischemia in the absence or presence of OX-A (40 µg/kg; i.p.). Data are mean ± SEM; *n* = 6 mice per group. Statistical analysis was performed by two-way ANOVA followed by the Bonferroni post hoc test. * *p* < 0.001 vs. sham-operated mice; ^#^ *p* < 0.005 vs. mice subjected to transient focal ischemia.

## References

[1] Kukkonen J.P., Holmqvist T., Ammoun S., Akerman K.E. (2002). Functions of the orexinergic/hypocretinergic system. Am. J. Physiol. Cell Physiol..

[2] Kukkonen J.P. (2012). Recent progress in orexin/hypocretin physiology and pharmacology. Biomol. Concepts.

[3] Nambu T., Sakurai T., Mizukami K., Hosoya Y., Yanagisawa M., Goto K. (1999). Distribution of orexin neurons in the adult rat brain. Brain Res..

[4] Peyron C., Tighe D.K., van den Pol A.N., de Lecea L., Heller H.C., Sutcliffe J.G., Kilduff T.S. (1998). Neurons containing hypocretin (orexin) project to multiple neuronal systems. J. Neurosci..

[5] Sakurai T., Amemiya A., Ishii M., Matsuzaki I., Chemelli R.M., Tanaka H., Williams S.C., Richardson J.A., Kozlowski G.P., Wilson S. (1998). Orexins and orexin receptors: A family of hypothalamic neuropeptides and G protein-coupled receptors that regulate feeding behavior. Cell.

[6] Armitage R. (2007). Sleep and circadian rhythms in mood disorders. Acta Psychiatr. Scand. Suppl..

[7] Kitamura E., Hamada J., Kanazawa N., Yonekura J., Masuda R., Sakai F., Mochizuki H. (2010). The effect of orexin-A on the pathological mechanism in the rat focal cerebral ischemia. J. Neurosci. Res..

[8] Sokołowska P., Urbańska A., Biegańska K., Wagner W., Ciszewski W., Namiecińska M., Zawilska J.B. (2014). Orexins protect neuronal cell cultures against hypoxic stress: An involvement of Akt signaling. J. Mol. Neurosci..

[9] Kong T., Qiu K., Liu M., Cheng B., Pan Y., Yang C., Chen J., Wang C. (2019). Orexin-A protects against oxygen-glucose deprivation/reoxygenation-induced cell damage by inhibiting endoplasmic reticulum stress-mediated apoptosis via the Gi and PI3K signaling pathways. Cell Signal..

[10] Chen H., Yoshioka H., Kim G.S., Jung J.E., Okami N., Sakata H., Maier C.M., Narasimhan P., Goeders C.E., Chan P.H. (2011). Oxidative Stress in Ischemic Brain Damage: Mechanisms of Cell Death and Potential Molecular Targets for Neuroprotection. Antioxid. Redox. Signal..

[11] Turunen P.M., Jäntti M.H., Kukkonen J.P. (2012). OX1 orexin/hypocretin receptor signalling through arachidonic acid and endocannabinoid release. Mol. Pharmacol..

[12] Cristino L., Luongo L., Imperatore R., Boccella S., Becker T., Morello G., Piscitelli F., Busetto G., Maione S., Di Marzo V. (2016). Orexin-A and Endocannabinoid Activation of the Descending Antinociceptive Pathway Underlies Altered Pain Perception in Leptin Signaling Deficiency. Neuropsychopharmacology.

[13] Mechoulam R., Ben-Shabat S., Hanus L., Ligumsky M., Kaminski N.E., Schatz A.R., Gopher A., Almog S., Martin B.R., Compton D.R. (1995). Identification of an endogenous 2-monoglyceride, present in canine gut, that binds to cannabinoid receptors. Biochem. Pharmacol..

[14] Sugiura T., Kondo S., Sukagawa A., Nakane S., Shinoda A., Itoh K., Yamashita A., Waku K. (1995). 2-Arachidonoylglycerol: A possible endogenous cannabinoid receptor ligand in brain. Biochem. Biophys. Res. Commun..

[15] Stella N., Schweitzer P., Piomelli D. (1997). A second endogenous cannabinoid that modulates long-term potentiation. Nature.

[16] Cristino L., Bisogno T., Di Marzo V. (2020). Cannabinoids and the expanded endocannabinoid system in neurological disorders. Nat. Rev. Neurol..

[17] Wang Q., Peng Y., Chen S., Gou X., Hu B., Du J., Lu Y., Xiong L. (2009). Pretreatment with electroacupuncture induces rapid tolerance to focal cerebral ischemia through regulation of endocannabinoid system. Stroke.

[18] Morello G., Imperatore R., Palomba L., Finelli C., Labruna G., Pasanisi F., Sacchetti L., Buono L., Piscitelli F., Orlando P. (2016). Orexin-A represses satiety-inducing POMC neurons and contributes to obesity via stimulation of endocannabinoid signaling. Proc. Natl. Acad. Sci. USA.

[19] Palomba L., Silvestri C., Imperatore R., Morello G., Piscitelli F., Martella A., Cristino L., Di Marzo V. (2015). Negative regulation of leptin-induced reactive oxygen species (ROS) formation by cannabinoid CB1 receptor activation in hypothalamic neurons. J. Biol. Chem..

[20] Chung H., Kim E., Lee D.H., Seo S., Ju S., Lee D., Kim H., Park S. (2007). Ghrelin inhibits apoptosis in hypothalamic neuronal cells during oxygen–glucose deprivation. Endocrinology.

[21] Palomba L., Amadori A., Cantoni O. (2007). Early release of arachidonic acid prevents an otherwise immediate formation of toxic levels of peroxynitrite in astrocytes stimulated with lipopolysaccharide/interferon-gamma. J. Neurochem..

[22] Cantoni O., Tommasini I., Cerioni L. (2008). The arachidonate-dependent survival signaling preventing toxicity in monocytes/macrophages exposed to peroxynitrite. Methods Enzymol..

[23] Bisogno T., Mahadevan A., Coccurello R., Chang J.W., Allarà M., Chen Y., Giacovazzo G., Lichtman A., Cravatt B., Moles A. (2013). A novel fluorophosphonate inhibitor of the biosynthesis of the endocannabinoid 2-arachidonoylglycerol with potential anti-obesity effects. Br. J. Pharmacol..

[24] Bülbül M., Tan R., Gemici B., Ongüt G., Izgüt-Uysal V.N. (2008). Effect of orexin-A on ischemia-reperfusion-induced gastric damage in rats. J. Gastroenterol..

[25] Butterick T.A., Nixon J.P., Billington C.J., Kotz C.M. (2012). Orexin A decreases lipid peroxidation and apoptosis in a novel hypothalamic cell model. Neurosci. Lett..

[26] Blankman J.L., Simon G.M., Cravatt B.F. (2007). A comprehensive profile of brain enzymes that hydrolyze the endocannabinoid 2-arachidonoylglycerol. Chem. Biol..

[27] Chanda P.K., Gao Y., Mark L., Btesh J., Strassle B.W., Lu P., Piesla M.J., Zhang M.-Y., Bingham B., Uveges A. (2010). Monoacylglycerol lipase activity is a critical modulator of the tone and integrity of the endocannabinoid system. Mol. Pharmacol..

[28] Schlosburg J.E., Blankman J.L., Long J.Z., Nomura D.K., Pan B., Kinsey S.G., Nguyen P.T., Ramesh D., Booker L., Burston J.J. (2010). Chronic mono-acylglycerol lipase blockade causes functional antagonism of the endocannabinoid system. Nat. Neurosci..

[29] Malagelada C., Xifro X., Minano A., Sabria J., Rodriguez-Alvarez J. (2005). Contribution of caspase-mediated apoptosis to the cell death caused by oxygenglucose deprivation in cortical cell cultures. Neurobiol. Dis..

[30] Ruscher K., Freyer D., Karsch M., Isaev N., Megow D., Sawitzki B., Priller J., Dirnagl U., Meisel A. (2002). Erythropoietin is a paracrine mediator of ischemic tolerance in the brain: Evidence from an in vitro model. J. Neurosci..

[31] Datta S.R., Brunet A., Greenberg M.E. (1999). Cellular survival: A play in three Akts. Genes Dev..

[32] Pearson G., Robinson F., Beers G.T., Xu B.E., Karandikar M., Berman K., Cobb M.H. (2001). Mitogen-activated protein (MAP) kinase pathways: Regulation and physiological functions. Endocr. Rev..

[33] Jiang Z., Zhang Y., Chen X., Lam P.Y., Yang H., Xu Q., Yu A.C. (2002). Activation of Erk1/2 and Akt in astrocytes under ischemia. Biochem. Biophys. Res. Commun..

[34] Chung H., Seo S., Moon M., Park S. (2008). Phosphatidylinositol-3-kinase/Akt/glycogen synthase kinase-3 beta and ERK1/2 pathways mediate protective effects of acylated and unacylated ghrelin against oxygen-glucose deprivation-induced apoptosis in primary rat cortical neuronal cells. J. Endocrinol..

[35] Zamzami N., Susin S.A., Marchetti P., Hirsch T., Gomez-Monterrey I., Castedo M., Kroemer G. (1996). Mitochondrial control of nuclear apoptosis. J. Exp. Med..

[36] Perez-Pinzon M.A., Xu G.P., Born J., Lorenzo J., Busto R., Rosenthal M., Sick T.J. (1999). Cytochrome c is released from mitochondria into the cytosol after cerebral anoxia or ischemia. J. Cereb. Blood Flow Metab..

[37] Liu Y.Y., Zhao Y.Y., Guo L. (2016). Effects of orexin A on glucose metabolism in human hepatocellular carcinoma in vitro via PI3K/Akt/mTOR-dependent and -independent mechanism. J. Mol. Cell. Endocrinol..

[38] Pasban-Aliabadi H., Esmaeili-Mahani S., Abbasnejad M. (2017). Orexin-A protects human neuroblastoma SH-SY5Y cells against 6-hydroxydopamine-induced neurotoxicity: Involvement of PKC and PI3K signaling pathways. Rejuvenation Res..

[39] Liu M.F., Xue Y., Liu C., Liu Y.H., Diao H.L., Wang Y., Pan Y.P., Chen L. (2018). Orexin-A exerts neuroprotective effects via OX1R in Parkinson’s disease. Front. Neurosci..

[40] Molina-Holgado F., Pinteaux E., Heenan L., Moore J.D., Rothwell N.J., Gibson R.M. (2005). Neuroprotective effects of the synthetic cannabinoid HU-210 in primary cortical neurons are mediated by phosphatidylinositol 3-kinase/AKT signaling. Mol. Cell Neurosci..

[41] Ozaita A., Puighermanal E., Maldonado R. (2007). Regulation of PI3K/Akt/GSK-3 pathway by cannabinoids in the brain. J. Neurochem..

[42] Yuan J., Yankner B.A. (2000). Apoptosis in the nervous system. Nature.

[43] Shi Y. (2001). A structural view of mitochondria-mediated apoptosis. Nat. Struct. Biol..

[44] Li P., Nijhawan D., Budihardjo I., Srinivasula S.M., Ahmad M., Alnemri E.S., Wang X. (1997). Cytochrome c and dATP-dependent formation of Apaf-1/caspase-9 complex initiates an apoptotic protease cascade. Cell.

[45] Kuida K., Haydar T.F., Kuan C.Y., Gu Y., Taya C., Karasuyama H., Su M.S., Rakic P., Flavell R.A. (1998). Reduced apoptosis and cytochrome c-mediated caspase activation in mice lacking caspase 9. Cell.

[46] Yoshida H., Kong Y.Y., Yoshida R., Elia A.J., Hakem A., Hakem R., Penninger J.M., Mak T.W. (1998). Apaf1 is required for mitochondrial pathways of apoptosis and brain development. Cell.

[47] Fujimura M., Morita-Fujimura Y., Murakami K., Kawase M., Chan P. (1998). H, Cytosolic redistribution of cytochrome c after transient focal cerebral ischemia in rats. J. Cereb. Blood Flow Metab..

[48] Hirai K., Sugawara T., Chan P.H., Basus V.J., James T.L., Litt L. (2002). Cytochrome c associated apoptosis during ATP recovery after hypoxia in neonatal rat cerebrocortical slices. J. Neurochem..

[49] Di Marzo V., Breivogel C.S., Tao Q., Bridgen D.T., Razdan R.K., Zimmer A.M., Zimmer A., Martin B.R. (2000). Levels, Metabolism, and pharmacological activity of anandamide in CB(1) cannabinoid receptor knockout mice: Evidence for non-CB(1), non-CB(2) receptor-mediated actions of anandamide in mouse brain. J. Neurochem..

[50] Liu J., Cinar R., Xiong K., Godlewski G., Jourdan T., Lin Y., Ntambi J.M., Kunos G. (2013). Monounsaturated fatty acids generated via stearoyl CoA desaturase-1 are endogenous inhibitors of fatty acid amide hydrolase. Proc. Natl. Acad. Sci. USA.

